# Effects of Fish Oil on Lipid Metabolism and Its Molecular Biological Regulators in Chronic Ethanol-Fed Rats

**DOI:** 10.3390/nu10070802

**Published:** 2018-06-22

**Authors:** Hsiao-Yun Wang, Hsiang-Chi Peng, Yi-Wen Chien, Ya-Ling Chen, Nien-Shan Lu, Suh-Ching Yang

**Affiliations:** 1 School of Nutrition and Health Sciences, Taipei Medical University, Taipei 11031, Taiwan; steventw11111@gmail.com (H.-Y.W.); hcpeng@tmu.edu.tw (H.-C.P.); ychien@tmu.edu.tw (Y.-W.C.); sandylu3339@gmail.com (N.-S.L.); 2 Research Center of Geriatric Nutrition, College of Nutrition, Taipei Medical University, Taipei 11031, Taiwan; 3 Graduate Institute of Metabolism and Obesity Sciences, Taipei Medical University, Taipei 11031, Taiwan; 4 Department of Nutrition and Health Sciences, Chang Gung University of Science and Technology, Taoyuan 33303, Taiwan; d507093001@tmu.edu.tw

**Keywords:** fish oil, adiponectin, fatty acid oxidation, ethanol-induced hepatic steatosis, Wistar rats

## Abstract

The purpose of this study was to clarify the hepatoprotective mechanisms of fish oil in ethanol-fed rats based on lipid metabolism. Thirty eight-week-old male Wistar rats were divided into six groups: C (control), CF25 (control diet with 25% fish oil substitution), CF57 (control diet with 57% fish oil substitution), E (ethanol-containing diet) group, EF25 (ethanol-containing diet with 25% fish oil substitution), and EF57 (ethanol-containing diet with 57% fish oil substitution) groups. All of the groups were pair-fed an isoenergetic diet based on E group. Rats were sacrificed after eight weeks. When compared with C group, the plasma aspartate transaminase (AST) activity and hepatic steatosis and inflammatory cell infiltration were significantly higher, while plasma adiponectin level and hepatic AMP-activated protein kinase α (AMPKα) protein expression was significantly lower in the E group. However, the hepatic damage, including steatosis and inflammation were ameliorated in the EF25 and EF57 groups. Moreover, mRNA levels of fatty acid-oxidative enzymes, such as medium-chain acyl-coenzyme A dehydrogenase (MCAD) and carnitine palmitoyltransferase I (CPT-1) were significantly elevated in the EF57 group than those in E group. Partial replacement with fish oil might improve the fatty acid oxidation by raising mRNA levels of downstream transcription factors, finally inhibit the ethanol-induced hepatic steatosis in rats.

## 1. Introduction

Alcohol abuse, alcohol dependence, and other alcohol-related health problems are important public health issues worldwide [1]. Chronic ethanol intake can cause fatty liver, hepatitis, cirrhosis, and even hepatoma. In Taiwan, hepatitis B and C are the main reasons for chronic liver disease. Furthermore, patients with hepatitis B or C may exacerbate their liver disease and increase the mortality rates by consuming alcohol [2]. Therefore, alcoholic liver disease (ALD) needs to garner greater attention in Taiwan.

Fish oil contains abundant amounts of *n*-3 polyunsaturated fatty acids (PUFAs), such as eicosapentaenoic acid (EPA) and docosahexaenoic acid (DHA), which were found to play important roles in lipid metabolism, antioxidative status, and immune function [3]. However, the effects of fish oil in ALD are still controversial. The possible reason is that fish oil was used as the only dietary source in many animal studies [4,5,6]. In our previous studies, we found that the replacement of olive oil with fish oil for (25% or 57%) in the diet significantly decreased plasma and hepatic TG and total cholesterol (TC) levels, thereby improving hepatic steatosis in rats fed with an ethanol-containing liquid diet for eight weeks [7]. However, the mechanism is still unknown.

Adiponectin is secreted from adipose tissues. It can combine with adiponectin receptor 2 (adipoR2) on hepatic membranes to activate AMP-activated protein kinase α (AMPKα) and NAD-dependent deacetylase sirtuin-1 (SIRT1). Usually, AMPKα and SIRT1 have cooperative interactions that affect hepatic lipid lipogenesis and lipolysis [8,9]. It was pointed out that plasma adiponectin levels and hepatic adipoR2 expression were reduced after chronic ethanol intake, which might inhibit the activation of hepatic AMPKα and SIRT1 [9]. The inactivation of hepatic AMPKα and SIRT1 caused the elevation of mRNA expressions of sterol response element-binding protein (SREBP)-1c, which is thought as a transcription factor of lipogenesis. SREBP-1c can induce the protein expression of enzymes that are related to fatty acid synthesis, such as fatty acid synthase (FAS), acetyl co(-enzyme) A carboxylase 1 (ACC1), and stearoyl coenzyme A desaturase (SCD)-1, to promote hepatic lipid synthesis and cause lipid accumulation [10]. Moreover, inactivation of hepatic AMPKα and SIRT1 also decreased peroxisome proliferator-activated receptor (PPAR)-α transcriptional activity and decreased medium-chain acyl-CoA dehydrogenase (MCAD) and acyl-CoA oxidase 1 (ACO1) mRNA levels that are related with lipolysis. In addition, inactivated AMPKα can decrease ACC1 phosphorylation, and then increase ACC1 activity to produce malonyl-CoA. Increased malonyl-CoA might decrease CPT-1 activity to suppress hepatic fatty acid oxidation [11]. Thus, reduced adiponectin levels might play a key role on hepatic lipogenesis and fatty acid oxidation after chronic ethanol consumption.

Therefore, we hypothesized that fish oil can improve chronic ethanol-induced steatosis by means of raising the adiponectin level, and then regulating its downstream transcription factors and enzymes that are related with lipogenesis or lipolysis. This animal study was performed to clarify the proposed hypothesis.

## 2. Materials and Methods

### 2.1. Animals

Thirty eight-week-old male Wistar rats (BioLasco Taiwan, Ilan, Taiwan) weighing about 250~280 g were used in this experiment. All of the rats were housed in individual cages in an animal room maintained at 22 ± 2 °C with 50~70% humidity and a 12-h light-dark cycle. All the rats were allowed free access to a standard rodent diet (LabDiet 5001 Rodent Diet; PMI Nutrition International, St. Louis, MO, USA) and water for one week of acclimation before the study. All of the procedures were approved by the Institutional Animal Care and Use Committee of Taipei Medical University.

### 2.2. Study Protocol

After one week of acclimation, rats were assigned to groups based on their plasma aspartate transaminase (AST) and alanine transaminase (ALT) activities, so there would be no differences in the plasma AST and ALT activities among groups. Rats were fed a control liquid diet or ethanol-containing liquid diet, and the fat composition of both diets was adjusted with 25% or 57% fish oil substituted for olive oil. Experimental models of ALD are commonly generated by feeding animals the Lieber-DeCarli liquid diet, in which fats in the diet are rich in monounsaturated fatty acids (MUFAs) and low in PUFAs [7]. Therefore, in this study, fish oil was used to substitute for part of the olive oil in the Lieber-DeCarli liquid diet according to MUFA/PUFA ratios. The MUFA/PUFA ratio of the diets without fish oil, and with 25% and 57% fish oil substitutions were 0.4, 0.7, and 1.5, respectively. Rats were divided into six groups: C (control diet), CF25 (control diet with 25% fish oil), CF57 (control diet with 57% fish oil), E (ethanol-containing diet), EF25 (ethanol-containing diet with 25% fish oil), and EF57 (ethanol-containing diet with 57% fish oil). The composition of the liquid diet was modified from Lieber-DeCarli liquid diet with isoenergetic pair-feeding based on E group [7]. After eight weeks, rats were anesthetized and sacrificed. Blood samples were collected in heparin-containing tubes and centrifuged (1730× *g* for 15 min at 4 °C) to obtain the plasma. All of the plasma samples were stored at −80 °C until being assayed. Liver tissues were rapidly excised and stored at −80 °C for further analysis.

### 2.3. Measurements and Analytical Procedures

#### 2.3.1. Plasma Biochemical Analyses

Plasma biochemical analyses, including AST and ALT activities, were conducted with the ADVIA^®^ 1800 Chemistry System (Siemens Healthcare Diagnostics, Eschborn, Germany).

#### 2.3.2. Plasma Adiponectin Concentration

The plasma adiponectin concentration was measured with an enzyme-linked immunosorbent assay (ELISA) kit (AssayMax rat adiponectin ELISA kit Assaypro, St. Charles, MO, USA). The optical density was read at 450 nm with a microplate reader (Molecular Devices, Sunnyvale, CA, USA).

#### 2.3.3. Histological Examinations

Histological observation of the liver used hematoxylin A-eosin (H&E) dye stain. In addition, semiquantitative histological evaluations of steatosis, inflammatory cell infiltration, and fibrosis were carried out according to a method that was described by Chiu et al. [12]

#### 2.3.4. Measurement of Hepatic TGs and TC

Hepatic lipids were extracted according to the method of Folch et al. [13]. Hepatic TG and TC concentrations were determined using diagnostic kits (Randox Laboratories, Antrim, UK).

#### 2.3.5. Hepatic adipoR2 and SIRT1 Protein Expressions

The method of crude extraction preparation from liver tissues was previously described [14]. Extracted crude protein (50 μg) was separated by 10% sodium dodecylsulfate polyacrylamide gel electrophoresis (SDS-PAGE). Proteins were electroblotted onto a polyvinylidene difluoride (PVDF) transfer membrane and then incubated with adipoR2 or SIRT1 antibodies, and β-actin or glyceraldehyde 3-phosphate dehydrogenase (GAPDH) was used as an internal control. Finally, the blot was treated with anti-mouse immunoglobulin G (IgG) or anti-rabbit IgG (Table 1), and specific bindings of antibodies were detected with a Western Lightning kit (Merck Millipore, Darmstadt, Germany). Bands were quantified using Image-Pro Plus 4.5 software.

#### 2.3.6. Hepatic AMPKα and Phosphorylated (p)-AMPKα Protein Expressions

Hepatic AMPKα and p-AMPKα were extracted, according to the method of Zhang et al., with minor modifications [15]. Extracted crude proteins (30 μg for AMPKα and 50 μg for p-AMPKα) were separated by 10% SDS-PAGE. Proteins were electroblotted onto a PVDF transfer membrane and then incubated with AMPKα, p-AMPKα, and GAPDH antibodies. The blot was treated with anti-mouse IgG and anti-rabbit IgG (Table 1). Methods of detection and quantification were the same as those that are described above.

#### 2.3.7. Total mRNA Extraction and Real-Time Quantitative Polymerase Chain Reaction (qPCR)

Total RNA was extracted with TRIzol^®^ reagent (Thermo Fisher Scientific, Waltham, MA, USA), according to the manufacturer’s instructions, with minor modifications. Total RNA isolated from the liver was reverse-transcribed with a RevertAid First Strand cDNA Synthesis kit (#K1621, Thermo Fisher Scientific). The resulting complementary (c)DNA was amplified in a 96-well PCR plate with SYBR Green/ROX qPCR Master Mix (2X) (Thermo Fisher Scientific) on an Applied Biosystems 7300 Real-Time PCR System (Thermo Fisher Scientific). Genes levels were normalized to that of β-actin, and the ratio to β-actin was calculated by setting the value of group C as 1. Information on primers is given in Table 2.

#### 2.3.8. Fatty Acid Composition

Crude lipids of liver tissues were extracted as described for the method of hepatic TGs. Crude lipids of red blood cells (RBCs), hepatic crude lipid extract, and lipids in the diets were extracted according to the method of Lee et al. [16]. Details of the fatty-acid methyl ester (FAME) analysis using a capillary gas chromatograph were the same as those given in Lee et al. [16] Fatty acid profiles were identified according to retention times of appropriate FAME standards. Composition data are expressed as weight percentages of total fatty acids.

#### 2.3.9. Statistical Analysis

Data are presented as the mean ± standard error of the mean (SEM). SAS software vers. 9.4 (SAS Institute, Cary, NC, USA) and Student’s *t*-test was used to determine statistical differences between C and E groups. A one-way analysis of variance (ANOVA), followed by Duncan’s new multiple-range test was used to determine statistical differences among the C, CF25, and CF57 groups and among the E, EF25, and EF57 groups. A two-way ANOVA was used to confirm the interaction between ethanol and fish oil. Statistical significance was assigned at the *p* < 0.05 level.

## 3. Results

### 3.1. Food Intake, Ethanol Consumption, Final Body Weight, and Relative Liver Weight

Food intake showed no differences among the six groups (C group: 74.97 ± 0.11 kcal/day, CF25 group: 75.08 ± 0.12 kcal/day, CF57 group: 75.04 ± 0.15 kcal/day, E group: 73.77 ± 2.41 kcal/day, EF25 group: 69.54 ± 1.97 kcal/day, and EF57 group: 70.31 ± 0.87 kcal/day). Average ethanol consumption levels in the E, EF25, and EF57 groups were 3.69 ± 0.12, 3.48 ± 0.10, and 3.52 ± 0.04 g/day, respectively. There was no difference in the ethanol intake among these groups.

Final body weights and relative liver weights are shown in Table 3. When compared to C group, the final body weight of E group was significantly lower (*p* < 0.05). However, there was no difference among the E, EF25, and EF57 groups. The relative liver weight of E group was significantly higher than that of the C group, but there were no differences among the ethanol-fed groups.

### 3.2. Plasma AST and ALT Activities and Hepatic Histopathology Scores

**Figure 1:**
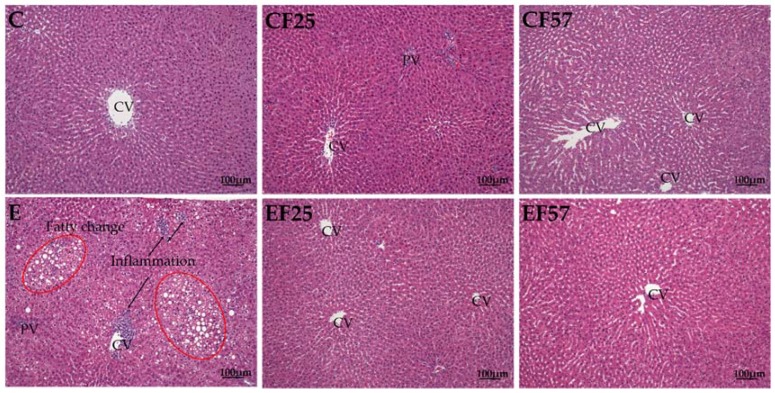
Effects of fish oil on liver pathology in rats with chronic ethanol feeding. CV, central vein; PV, portal vein. C, control group; CF25, control diet with fish oil substituted for 25% of olive oil; CF57, control diet with fish oil substituted for 57% of olive oil; E, ethanol group; EF25, alcohol-containing diet with fish oil substituted for 25% of olive oil; EF57, alcohol-containing diet with fish oil substituted for 57% of olive oil. hematoxylin A & eosin (H&E) staining showed hepatocyte degeneration and necrosis accompanied by inflammatory cell infiltration (arrow) in E group. Moreover, fatty changes (red circle) were also found in E group.

Plasma AST and ALT activities in E group were significantly higher than those in C group after eight weeks of feeding (*p* < 0.05). However, plasma AST activities of the EF25 and EF57 groups were significantly lower than that of E group (Table 3). Hepatic steatosis and inflammatory cell infiltration in E group showed significant elevation, while those scores were significantly lower in the EF25 and EF57 groups (Table 4). According to the hepatic histopathology, steatosis and inflammatory cell infiltration were observed in E group (Figure 1).

### 3.3. Hepatic TG and TC Levels

Hepatic TG and TC levels in E group were significantly higher than those in C group. However, hepatic TG levels were significantly decreased in the EF25 and EF57 groups. There were no differences in hepatic TC levels among E, EF25, and EF57 groups (Table 3).

### 3.4. Plasma Adiponectin Levels, and Hepatic AdipoR2, AMPKα, p-AMPKα, and SIRT1 Protein Expressions

**Figure 2:**
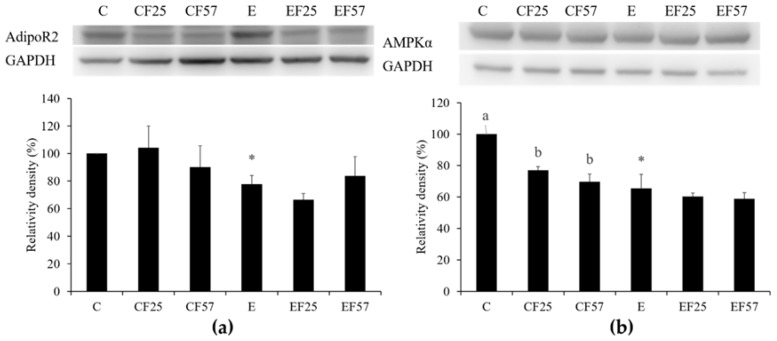

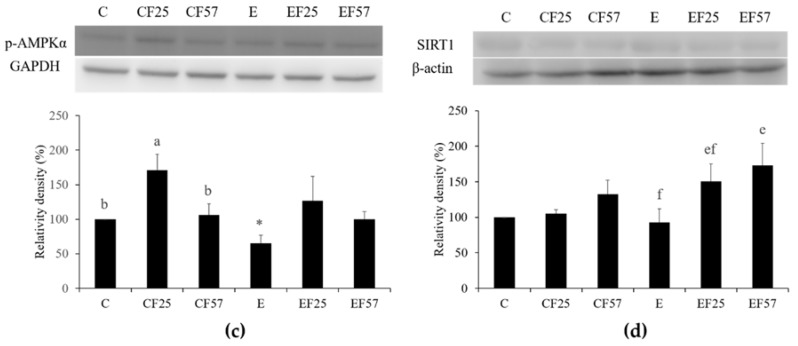
Effects of fish oil on hepatic adiponectin receptor 2 (adipoR2), AMP-activated protein kinase-α (AMPKα), phosphorylated (p)-AMPKα, and NAD-dependent deacetylase sirtuin-1 (SIRT1) protein expressions in rats with chronic ethanol feeding. Western blots analysis of (**a**) adipoR2, (**b**) AMPKα, (**c**) p-AMPKα, and (**d**) SIRT1 protein expressions. β-Actin or glyceraldehyde 3-phosphate dehydrogenase (GAPDH) was used as an internal control. Quantitative analysis of protein levels and the ratio to each internal control was calculated by setting the value of group C as 1. Values are expressed as the mean ± SEM. An asterisk (*) shows a significant difference between C and E groups (*p* < 0.05). Means with different superscript letters shows a significant difference (a,b) among C, CF25, and CF57 groups (*p* < 0.05). Means with different superscript letters shows a significant difference (e,f) among E, EF25, and EF57 groups (*p* < 0.05).

When compared with C group, the plasma adiponectin level was significantly lower in E group. However, EF57 group showed a significantly higher plasma adiponectin level when compared to E group (Table 3). In E group, hepatic AdipoR2, AMPKα, and p-AMPKα protein expressions were significantly lower than those in group C (Figure 2a–c). Hepatic SIRT1 protein expression showed no difference between C and E groups, but it was significantly enhanced in EF57 group as compared to E group (Figure 2d). There were no differences among E, EF25, and EF57 groups in hepatic AdipoR2, AMPKα, or p-AMPKα protein expressions Figure 2a–c).

### 3.5. Hepatic Fatty Acid Metabolism-Related Gene mRNA Levels

As Table 5 shows, mRNA level of SREBP-1c as the transcription factor of fatty acid synthesis showed no differences between in C and E groups. Furthermore, mRNA levels of FAS and SCD-1 as the target genes of SREBP-1c also showed no differences between C and E groups. SREBP-1c, FAS, and SCD-1 mRNA levels did not differ among the E, EF25, and EF57 groups. On the other hand, there was no change in PPAR-α mRNA level as a transcription factor of fatty acid oxidation between C and E groups. However, when compared with C group, PPAR-α mRNA level was significantly increased in the CF25 and CF57 group. However, there was no difference in PPAR-α mRNA level among E, EF25, and EF57 groups. Representing a target gene of PPAR-α, the MCAD mRNA level did not differ between C and E groups, while it was significantly higher in the EF57 group that of E group. The mRNA level of ACO-1, which is another target gene of PPAR-α, did not change among all of the groups. The mRNA level of CPT-1, which is an enzyme of fatty acid oxidation, showed no difference between the C and E groups. However, the CPT-1 mRNA level in EF57 group was significantly higher when compared with E group.

### 3.6. RBC Membranes and Hepatic Fatty Acid Composition

According to the fatty acid composition of RBC membranes, C16:0 and C18:1 levels were significantly higher and the C18:0 level was significantly lower in the E group than in C group (Table 6). However, the C18:1 level of RBC membranes was significantly lower and the C18:0 level of RBC membranes was significantly higher in EF57 group than in E group (Table 6). There was no difference in the ratio of *n*-6/*n*-3 among all of the groups (Table 6).

On the other hand, C16:0 and C18:3 (ALA) levels of hepatocytes were significantly lower and the C18:1 level was significantly higher in E group than in C group (Table 7). However, C18:1, C18:2, and C20:4 (AA) levels of hepatocytes were significantly lower and C20:5 (EPA), C22:5 (DPA), and C22:6 (DHA) levels of hepatocytes were significantly higher in EF25 and EF57 groups than in E group (Table 7). There was no change in the ratio of *n*-6/*n*-3 in hepatocytes between C and E groups; however, the ratio was significantly decreased not only in CF25 and CF57 groups, but also in EF25 and EF57 groups when compared with C and E groups, respectively (Table 7).

## 4. Discussion

### 4.1. Body Weight and Liver Damage

In the present study, rats fed with ethanol without fish oil showed the significantly lower body weight and the significantly heavier related liver weight (Table 3). ALD patients are reported to have a malnutrition problem, which causes muscle and body weight loss [17]. Moreover, 90~95% of ALD patients are with hepatomegaly [18].

Hepatic steatosis and the inflammatory cell infiltration score in the E group were significantly higher as compared to those of C group (Table 4). Plasma AST and ALT activities were also significantly increased in the E group. Chiu et al. reported that simultaneously evaluated plasma AST and ALT activities and hepatic histopathological scores could reflect the actual liver damage status [12]. Based on the data of liver damage, it was definite that the animal model of alcoholic liver damage was successfully established in this study.

### 4.2. Ethanol, Fish Oil and Hepatic Lipid Profiles

It was pointed out that chronic ethanol consumption might contribute to adipose tissue lipolysis and increase plasma free fatty acid influx into the liver [19]. At the same time, increased hepatic glycerol-3-phosphate acyltransferase 3 (GPAT3) mRNA might lead to the increased lipogenesis and TG levels [19]. It was also mentioned that acetaldehyde, which is produced by ethanol oxidation through the ADH (alcohol dehydrogenase) and CYP2E1 pathway, might inhibit hepatic lipolysis by downregulating PPAR-α transcriptional activity and decreasing levels of lipolysis-related genes [20]. In the present study, we found that the hepatic TG level was significantly increased in E group after eight weeks of ethanol intake (Table 3). These results were similar to the previous study [7,12].

Results also showed that the hepatic TG level was alleviated by dietary fish oil replacement (Table 3). A previous study indicated that after feeding on a fish oil-containing diet (10% *w*/*w*; EPA:DHA = 4:3) for 16 weeks, hepatic SREBP-1c expression and the downstream gene mRNA level significantly decreased in SD rats [21]. In addition, this diet might increase expressions of hepatic lipolysis-related genes, and promote lipid exclusion from the liver [21]. Moreover, the hepatic TG level was decreased, and steatohepatitis was improved in obese mice fed with an *n*-3 PUFA-abundant diet [22]. However, in the 1990s, Nanji et al. indicated that fish oil might cause more-serious steatosis and liver damage [4,5,6]. The reason for the inconsistent results may be that Nanji et al.’s study used fish oil was the only one dietary fat source and excessive *n*-3 PUFAs might induce oxidative stress and lead to liver damage. Therefore, we concluded that the dietary fat combination and fatty acid composition might be the key point for improving ethanol-induced liver damage in rats [7].

### 4.3. Ethanol, Fish Oil, and the Adiponectin-AMPKα-SIRT1 Pathway

Results showed that after eight weeks of ethanol intake, the plasma adiponectin level and hepatic adipoR2, AMPKα, and p-AMPKα protein expressions had significantly decreased (Table 3, Figure 2a–c), while hepatic SIRT1 protein expression exhibited a downward trend (Figure 2d). It was pointed out that rats with chronic ethanol intake had significantly lower plasma adiponectin levels [8]. It was also reported that hepatic adipoR2 protein expression was significantly reduced in mice that were chronically fed with ethanol [23]. It has been indicated that AMPKα and p-AMPKα protein expressions were reduced that decreased ACC1 phosphorylation, in addition, the hepatic SIRT1 mRNA level and protein expression were reduced in rats fed with ethanol for four weeks [24]. Therefore, we speculated that the plasma adiponectin level and hepatic adipoR2 expression might have been reduced and then led to decrease AMPKα expression to promote hepatic lipogenesis in rats after eight weeks of ethanol intake. On the other hand, it was indicated that not only hepatic SIRT1 expression but also activity was affected by the chronic ethanol intake [25]. In this study, the reduction of hepatic SIRT1 expression was not obvious in E group. Therefore, SIRT1 expression and activity should be examined at the same time to determine the actual effect of chronic ethanol intake on SIRT1.

When compared to E group, the plasma adiponectin level and hepatic SIRT1 protein expression were significantly increased in the EF57 group (Table 3, Figure 2d). In non-alcoholic fatty liver disease (NAFLD) patients and NAFLD model rats, fish oil supplementation increased the plasma adiponectin level [26,27]. When mice were intravenously injected with adiponectin (30 μg/day) in chronic ethanol intake, the AMPKα signaling pathway was activated for inhibiting hepatic steatosis [28]. In addition, rat H4IIEC3 cells exhibited increased SIRT1 and AMPKα expressions after treating with adiponectin for 24 h [29]. Thus, we supposed that the plasma adiponectin level was increased, which induced the hepatic SIRT1 expression when dietary olive oil was partially substituted with fish oil in rats after eight weeks of ethanol intake.

### 4.4. Ethanol, Fish Oil and the SREBP-1c Pathway

SREBP-1c is a transcription factor of lipogenesis, and its target enzymes include FAS and SCD-1, etc. In the present study, SREBP-1c, FAS, and SCD-1 mRNA levels only upward trends were observed in E group (Table 5). Chronic ethanol intake decreased SIRT1 expression and reduced SREBP-1c deacetylation, and then promoted SREBP1-c transcriptional activity. SREBP-1c might increase FAS and SCD-1 mRNA levels to cause hepatic steatosis [9]. Although lipogenic genes only had upward trends in the E group, hepatic steatosis was found in E group according to the hepatic histopathology. We speculated that other factors that improve lipogenesis by ethanol intake might be involved, such as hepatic lipoprotein secretion ability, the NAD+/NADH ratio, G3PD activity, etc.

In the present study, SREBP-1c, FAS, and SCD-1 mRNA levels showed no differences among the E, EF25, and EF57 groups (Table 5). Nakatani et al. indicated that after giving different amounts of fish oil to mice with NAFLD, protein expression of SREBP-1c had an upward trend in mice when 10~20% of total calories were supplied as fish oil. However, protein expression of SREBP-1c and mRNA levels of FAS and SCD-1 significantly decreased in mice that were provided with fish oil as 40~60% of total calories [30]. Fish oil supplementation in EF25 and EF57 groups were 6% and 14% of total calories, respectively. Thus, we supposed that the amount of fish oil was too low to showing the inhibiting potential for lipogenesis. However, based on our hepatic histopathology scores, 25% or 57% fish oil substitution for olive oil truly improved hepatic lipid accumulation.

### 4.5. Ethanol, Fish Oil and the PPAR-α Pathway

Under normal conditions, hepatic AMPKα and SIRT1 activate PGC-1α to promote PPAR-α transcriptional activity and increase MCAD and ACO1 mRNA levels [9]. On the other hand, AMPKα also increases ACC1 phosphorylation to reduce the malonyl-CoA level and increase CPT-1 protein expression to promote lipolysis [12]. In this study, MCAD, ACO1, and CPT-1 mRNA were not changed in E group (Table 5). The factors that the mechanisms of chronic ethanol ingestion were complicated and interrelated, thus, we strongly suggested that the activities of those enzymes have to be measured in future.

MCAD and CPT-1 mRNA levels in EF57 group were significantly increased when compared to those of group E (Table 5). After fish oil supplementation, mRNA levels of PPAR-α, MCAD, ACO1, and CPT-1 were significantly increased in acute ethanol-treated mice [11]. In an NAFLD gerbil model, EPA intervention significantly increased the hepatic PPAR-α mRNA level and decreased the hepatic TG level [31]. Thus, we speculated that 57% fish oil substitution for olive oil could increase the MCAD and CPT-1 mRNA levels to promote hepatic lipolysis in this study, although the PPAR-α mRNA level only showed the increasing trend.

### 4.6. Ethanol, Fish Oil and RBC Membrane and Hepatic Fatty Acid Compositions

Results showed that C16:0, C18:1, and total *n*-6 fatty acids were significantly increased, but C18:0 was significantly decreased in RBC membranes in E group (Table 6). We also found that hepatic C16:0 and C18:3 (ALA) decreased and C18:1 significantly increased in E group (Table 7). The change of C16:0 and C18:1 in RBC membrane and hepatocytes was totally opposite in rats that were fed with ethanol for eight weeks. The fatty acid composition of RBC membrane can represent the whole-body circulation during 2~3 months, while as the fatty acid composition in specific cells can reflect the situation of the specific tissue per se. Many factors can regulate the fatty acid composition in RBC membrane, including dietary pattern, metabolic stress, medicines, or chronic diseases, etc. The saturation index (SI) in erythrocytes membrane, which is calculated by the C18:0/C18:1 ratio, was significantly decreased in cancer patients [32,33]. Some previous studies also showed the changes of fatty acids composition in specific cell membranes and indicated that C16:0 promoted inflammation and apoptosis in white adipose tissues. Moreover, 3T3-L1 adipocytes that were treated with C16:0 increased protein kinase C (PKC), nuclear factor (NF)-κB, and mitogen-activated protein kinase (MAPK) expressions to promote cytokine secretion [34]. Furthermore, hepatic C16:0 content was related with hepatic steatosis or inflammation and hepatic SCD-1 could convert C16:0 to C18:1 to reduce the toxicity of C16:0 [35,36]. Therefore, we speculated that the changes of C16:0, C18:0, and C18:1 might play an important role in the progression of ALD. We require more clarification in future study.

Only in EF57 group, C16:0 was significantly decrease in RBC membrane (Table 6). In hepatocytes, C20:5, C22:5, C22:6, and total *n*-3 fatty acids were significantly increased, and C18:1, C18:2, C20:4, total *n*-6, and the *n*-6/*n*-3 ratio were significantly decreased in the EF25 and EF57 groups (Table 7). These results showed that dietary fish oil substituted for olive oil mainly regulated the fatty acid composition in hepatocytes membranes rather than erythrocytes membranes, which might be related with the severity of liver damage that is caused by chronic ethanol intake.

In order to clarify the relationship between fatty acid composition and liver damage, we also analyzed the correlations of hepatic *n*-3, *n*-6 fatty acids and the *n*-6/*n*-3 ratio with the histopathology scores and factors of lipid metabolism (Table 8). Our data showed that hepatic *n*-3 fatty acids were negatively correlated with inflammatory cell infiltration (*r =* −0.44613, *p =* 0.00135), and were positively correlated with lipid metabolism factors, SIRT1 expression (*r =* 0.46795, *p =* 0.004), and MCAD (*r =* 0.43524, *p =* 0.008) and CPT-1 (*r =* 0.37398, *p =* 0.0418) mRNA levels. On the contrary, *n*-6 fatty acids were positively correlated with steatosis (*r =* 0.37631, *p =* 0.0404) and inflammatory cell infiltration (*r =* 0.50184, *p =* 0.0047) and negatively correlated with plasma adiponectin levels (*r =* −0.35711, *p =* 0.0325). In addition, the *n*-6/*n*-3 ratio was positively correlated with inflammatory cell infiltration (*r =* 0.50691, *p =* 0.0043) and negatively correlated with the plasma adiponectin level (*r =* −0.34411, *p =* 0.0399) and SIRT1 expression (*r =* −0.44387, *p =* 0.0067). Taken together, we speculated that increased *n*-3 fatty acids and decreased *n*-6 fatty acids were strongly correlated with ameliorating alcoholic liver damage.

## 5. Conclusions

**Figure 3:**
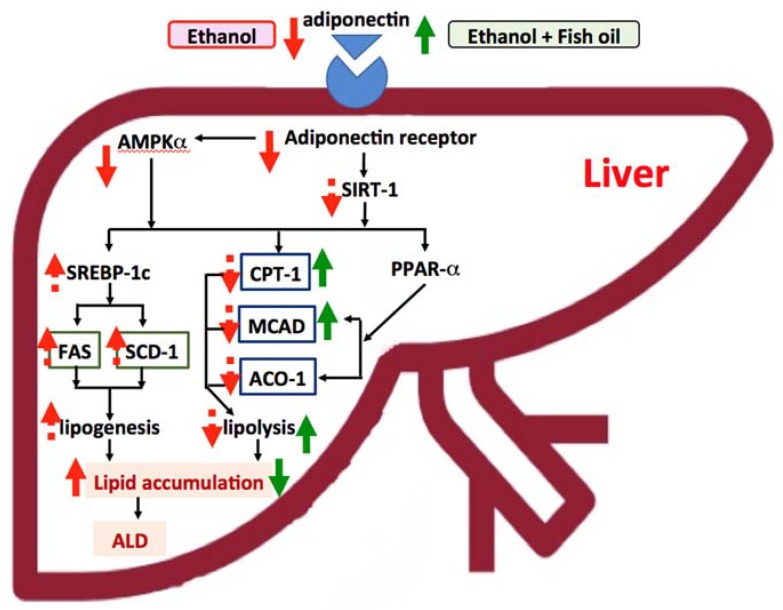
Effects of fish oil on improving alcoholic liver disease (ALD). The effects for ethanol are presented as red-dotted and solid-line arrows, and dotted lines are presented as up- or down-trending effects. The effects for ethanol with fish oil are presented as green solid-line arrows. (1) In this study, we indicated that chronic ethanol intake decreased the plasma adiponectin level and hepatic adiponectin receptor 2 (adipoR2) protein expression and then possibly reduced AMP-activated protein kinase α (AMPKα). Decreased AMPKα might increase sterol response element-binding protein (SREBP)-1c, fatty acid synthase (FAS), and stearoyl coenzyme A desaturase (SCD)-1 mRNA levels and improve hepatic fatty acid synthesis (only an upward trend). The change of carnitine palmitoyl transferase I (CPT1), medium-chain acyl-coenzyme A dehydrogenase (MCAD), and acyl-CoA oxidase 1 (ACO1) mRNA levels were not observed in this study. (2) With 57% fish oil substitution for olive oil, the plasma adiponectin level was significantly increased and the mRNA levels of downstream enzymes, such as hepatic CPT1 and MCAD were also elevated, which might improve the lipolysis and ameliorate hepatic steatosis in rats fed with ethanol for eight weeks. SIRT1, NAD-dependent deacetylase sirtuin-1.

As shown in Figure 3, hepatosteatosis formation might be due to due to the low plasma adiponectin level and the hepatic adiponectin receptor, which continuously regulated the downstream factors, such as protein expressions of AMPKα and p-AMPKα in chronic ethanol-fed rats. However, using 57% fish oil substitution for olive oil, inhibited the reduction of plasma adiponectin and elevated hepatic CPT-1 and MCAD mRNA levels, which improved the fatty acid oxidation and further prevented ethanol-induced hepatic steatosis in rats. More detailed studies are needed to elucidate the mechanisms for explaining the ameliorating effects of fish oil on ALD.

## Figures and Tables

**Table 1:** Antibodies used for Western blotting.

	Antibody (Ab)	Ab Type	Product No.	Source
Primary antibody	adipoR2	monoclonal	sc-514045	Santa Cruz Biotechnology
	SIRT1	monoclonal	#9475	Cell Signaling Technology
	AMPKα	polyclonal	#2532	Cell Signaling Technology
	phospho-AMPKα	monoclonal	#2535	Cell Signaling Technology
Internal	β-actin	monoclonal	MAB1501	Millipore
control	GAPDH	monoclonal	#97166	Cell Signaling Technology
Secondary antibody	anti-mouse IgG		AP124P	Millipore
	anti-rabbit IgG		111-035-003	Jackson ImmunoResearch Laboratories

**Table 2:** Primers used for the quantitative polymerase chain reaction.

	Forward 5′→3′	Reverse 5′→3′	GenBank No.
SREBP-1c	AGGAGGCCATCTTGTTGCTT	GTTTTGACCCTTAGGGCAGC	XR_001840090.1
FAS	CGGCGTGTGATGGGGCTGGTA	AGGAGTAGTAGGCGGTGGTGTAGA	X62889.1
SCD1	GTTGGGTGCCTTATCGCTTTCC	CTCCAGCCAGCCTCTTGTCTAC	XM_006231433.2
PPARα	CGGGTCATACTCGCAGGAAA	AAGCGTCTTCTCAGCCATGC	XM_017594681.1
MCAD	GCGGGCATTAAGACCAAAGC	GCCTTTCCCCCGTTGGTTAT	XM_021158408.1
ACO1	TTCAAGACAAAGCCGTCCAA	TGCTCCCCTCAAGAAAGTCC	XM_021635981.1
CPT-1	GCATCCCAGGCAAAGAGACA	CGAGCCCTCATAGAGCCAGA	JN960994.1
β-Actin	CACCAGTTCGCCATGGATGACGA	CCATCACACCCTGGTGCCTAGGGC	XM_021163894.1

**Table 3:** Effects of fish oil on the final body weight, relative liver weight, plasma alanine transaminase (ALT) and aspartate transaminase (AST) activities, plasma adiponectin, and hepatic lipid profiles levels in rats with chronic ethanol feeding ^1,2^.

			F25	F57	Ethanol × Fish Oil
Final body weight (g)	C	425.2 ± 4.4 ^c^	440.2 ± 4.7 ^a^	437.0 ± 10.7 ^b^	0.74
	E	392.0 ± 11.9 *	379.3 ± 12.9	398.7 ± 6.0	
Relative liver weight (%) ^3^	C	2.2 ± 0 ^b^	2.3 ± 0 ^b^	2.5 ± 0 ^a^	0.4533
	E	2.9 ± 0 *	3.1 ± 0.1	3.2 ± 0.2	
Plasma ALT activity (U/L)	C	33.7 ± 1.9 ^b^	39.7 ± 6.9 ^a,b^	41.8 ± 6.7 ^a^	0.1781
	E	78.0 ± 15.0 *	81.2 ± 21.4	66.2 ± 13.4	
Plasma AST activity (U/L)	C	75.8 ± 5.0 ^b^	77.2 ± 1.2 ^b^	91.5 ± 3.2 ^a^	0.0003
	E	151.5 ± 10.0 *^,e^	122.0 ± 9.8 ^f^	131.2 ± 16.8 ^f^	
Hepatic TGs (mg TGs/g liver)	C	9.05 ± 0.64	10.00 ± 0.68	12.26 ± 1.25	<0.0001
	E	15.51 ± 0.42 *^,e^	11.61 ± 1.06 ^f^	8.93 ± 0.52 ^g^	
Hepatic TC (mg TC/g liver)	C	13.10 ± 0.70 ^b^	14.60 ± 1.00 ^a,b^	17.10 ± 1.10 ^a^	0.8515
	E	17.90 ± 0.90 *	19.00 ± 1.30	20.50 ± 1.40	
Plasma adiponectin (μg/mL)	C	12.2 ± 1.0 ^a^	13.9 ± 0.9 ^a,b^	16.5 ± 2.0 ^b^	0.947
	E	5.1 ± 1.3 *^,f^	7.4 ± 1.3 ^e,f^	10.3 ± 1.5 ^e^	

^1^ Values are expressed as the mean ± SEM. An asterisk (*) shows a significant difference between the C and E groups (*p* < 0.05). Means with different superscript letters (^a,b,c^) shows a significant difference among the C, CF25, and CF57 groups (*p* < 0.05). Means with different superscript letters shows a significant difference (^e,f,g^) among the E, EF25, and EF57 groups (*p* < 0.05). ^2^ C, control group; CF25, control diet with fish oil substituted for 25% of olive oil; CF57, control diet with fish oil substituted for 57% of olive oil; E, ethanol group; EF25, alcohol-containing diet with fish oil substituted for 25% of olive oil; EF57, alcohol-containing diet with fish oil substituted for 57% of olive oil. ^3^ Relative liver weight: (liver weight/body weight) × 100%. TGs: triglycerides; TC: total cholesterol.

**Table 4:** Effects of fish oil on hepatic histopathology in rats with chronic ethanol feeding ^1,2^.

			F25	F57	Ethanol × Fish Oil
Steatosis	C	0.2 ± 0.2	0.2 ± 0.2	0.4 ± 0.2	0.0194
	E	2.2 ± 0.2 *^,e^	1.0 ± 0.4 ^f^	0.6 ± 0.4 ^f^	
Inflammatory cell infiltration	C	0.6 ± 0.2	0.4 ± 0.2	0 ± 0	0.042
	E	2.8 ± 0.2 *^,e^	1.2 ± 0.4 ^f^	0.8 ± 0.5 ^f^	
Fibrosis	C	0 ± 0	0 ± 0	0 ± 0	0.3157
	E	0.6 ± 0.2	0.2 ± 0.2	0.2 ± 0.2	

^1^ Values are expressed as the mean ± SEM. An asterisk (*) shows a significant difference between C and E groups (*p* < 0.05). Means with different superscript letters shows a significant difference (^e,f^) among the E, EF25, and EF57 groups (*p* < 0.05). ^2^ Details are the same as those described in Table 3.

**Table 5:** Effects of fish oil on hepatic fatty acid metabolism-related gene mRNA levels in rats with chronic ethanol feeding ^1,2,3^.

mRNA Levels			F25	F57	Ethanol × Fish Oil
SREBP-1c	C	1.00 ± 0.30	1.54 ± 0.29	1.90 ± 0.38	0.5486
	E	1.55 ± 0.23	1.60 ± 0.17	1.92 ± 0.36	
FAS	C	1.00 ± 0.29	1.48 ± 0.19	1.87 ± 0.50	0.2566
	E	1.57 ± 0.27	1.50 ± 0.2	1.53 ± 0.12	
SCD-1	C	1.00 ± 0.22	1.34 ± 0.19	1.37 ± 0.22	0.7705
	E	1.41 ± 0.29	1.48 ± 0.26	1.78 ± 0.19	
PPARα	C	1.00 ± 0.28 ^b^	2.03 ± 0.24 ^a^	2.11 ± 0.28 ^a^	0.7043
	E	1.39 ± 0.40	2.15 ± 0.42	2.03 ± 0.19	
MCAD	C	1.00 ± 0.14	0.96 ± 0.14	1.20 ± 0.14	0.2107
	E	0.83 ± 0.12 ^f^	1.13 ± 0.05 ^e,f^	1.63 ± 0.33 ^e^	
ACO1	C	1.00 ± 0.51	1.32 ± 0.15	1.25 ± 0.14	0.9118
	E	0.93 ± 0.18	1.41 ± 0.32	1.40 ± 0.21	
CPT-1	C	1.00 ± 0.21	1.16 ± 0.17	1.37 ± 0.43	0.5085
	E	0.99 ± 0.13 ^f^	1.09 ± 0.12 ^f^	1.74 ± 0.14 ^e^	

^1^ Values are expressed as the mean ± SEM. Means with different superscript letters shows a significant difference (^a,b^) among C, CF25, and CF57 groups (*p* < 0.05). Means with different superscript letters shows a significant difference (^e,f^) among E, EF25, and EF57 groups (*p* < 0.05). ^2^ Details are the same as those described in Table 3. ^3^ Comparative quantification of each gene was normalized to β-actin using the 2^−ΔΔCt^ method. SREBP, sterol response element-binding protein; FAS, fatty acid synthase; SCD, stearoyl coenzyme A desaturase; PPAR, peroxisome proliferator-activated receptor; MCAD, medium-chain acyl-coenzyme A dehydrogenase; ACO1, acyl-CoA oxidase 1; CPT, carnitine palmitoyl transferase.

**Table 6:** Effects of fish oil on the red blood cell membrane fatty acid composition in rats with chronic ethanol feeding ^1,2^.

Fatty Acid (%)	C	CF25	CF57	E	EF25	EF57	Ethanol × Fish Oil
C16:0	48.87 ± 2.35 ^b^	54.69 ± 1.4 ^a^	51.75 ± 1.42 ^a,b^	58.68 ± 2.89 *^,e^	56.83 ± 3 ^e^	48.39 ± 0.46 ^f^	0.0072
C18:0	21.72 ± 0.44	21.02 ± 0.69	20.59 ± 0.81	3.73 ± 4.09 *^,f^	11.12 ± 5.45 ^e,f^	21.71 ± 0.39 ^e^	0.0034
C18:1 (OA, *n*-9)	15.59 ± 0.53	14.37 ± 0.8	15.01 ± 0.51	21.41 ± 1.28 *^,e^	19.19 ± 1.25 ^e,f^	17.23 ± 0.16 ^f^	0.0752
C18:2 (LA, *n*-6)	5.01 ± 0.39	4.31 ± 0.64	4.63 ± 0.68	6.03 ± 0.45	5.26 ± 0.62	4.76 ± 0.17	0.5821
C18:3 (ALA, *n*-3)	0.82 ± 0.29	0.76 ± 0.21	1.1 ± 0.23	1.32 ± 0.13	1.26 ± 0.22	1.15 ± 0.08	0.4043
C20:4 (AA, *n*-6)	1.67 ± 0.48	0.76 ± 0.21	1.35 ± 0.5	1.99 ± 0.45 ^e^	0.96 ± 0.17 ^f^	1.19 ± 0.09 ^f^	0.7422
C20:5 (EPA, *n*-3)	1.87 ± 1.34	0.41 ± 0.12	0.67 ± 0.19	1.04 ± 0.24	0.82 ± 0.3	0.98 ± 0.14	0.4347
C22:5 (DPA, *n*-3)	0.07 ± 0.05	0.11 ± 0.05	0.33 ± 0.14	0.05 ± 0.06 ^e,f^	0.03 ± 0.03 ^f^	0.17 ± 0.04 ^e^	0.5656
C22:6 (DHA, *n*-3)	1.29 ± 0.94	0.4 ± 0.1	0.73 ± 0.24	0.84 ± 0.09 ^e^	0.72 ± 0.15 ^e,f^	0.49 ± 0.07 ^f^	0.5699
SFAs	71.48 ± 2.33	76.95 ± 2.05	73.72 ± 2.29	64.16 ± 1.51 *^,f^	69.37 ± 2.62 ^e^	71.71 ± 0.56 ^e^	0.2471
MUFAs	15.59 ± 0.53	14.37 ± 0.8	15.01 ± 0.51	21.41 ± 1.28 *^,e^	19.19 ± 1.25 ^e,f^	17.23 ± 0.16 ^f^	0.0752
PUFAs	12.94 ± 2.66	8.68 ± 1.32	11.27 ± 2.21	14.44 ± 0.48 ^e^	11.44 ± 1.56 ^f^	11.06 ± 0.47 ^f^	0.6211
Total *n*-3	4.06 ± 2.04	1.68 ± 0.39	2.83 ± 0.68	3.26 ± 0.45	2.83 ± 0.65	2.79 ± 0.25	0.539
Total *n*-6	3.87 ± 0.34	2.69 ± 0.46	3.82 ± 0.87	5.14 ± 0.21 *^,e^	3.35 ± 0.5 ^f^	3.51 ± 0.18 ^f^	0.2175
*n*-6/*n*-3	1.46 ± 0.26	1.84 ± 0.31	1.4 ± 0.08	1.86 ± 0.52	1.26 ± 0.09	1.29 ± 0.08	0.1641

^1^ Values are expressed as the mean ± SEM. An asterisk (*) shows a significant difference between C and E groups (*p* < 0.05). Means with different superscript letters shows a significant difference (^a,b^) among C, CF25, and CF57 groups (*p* < 0.05). Means with different superscript letters shows a significant difference (^e,f^) among E, EF25, and EF57 groups (*p* < 0.05). ^2^ Crude lipids of red blood cells (RBCs) were extracted according to the method of Lee et al. [16]. Details of the fatty-acid methyl ester (FAME) analysis using a capillary gas chromatograph were same as those given in Lee et al. [16]. Details are the same as those described in Table 3. OA, oleic acid; LA, linoleic acid; ALA, alpha-linolenic acid; AA, arachidonic acid; EPA, eicosapentaenoic acid; DPA, all-cis-7,10,13,16,19-docosapentaenoic acid; DHA, docosahexaenoic acid; SFAs, saturated fatty acids; PUFAs, polyunsaturated fatty acids; MUFAs, monounsaturated fatty acids.

**Table 7:** Effects of fish oil on hepatic cell membrane fatty acid compositions in rats with chronic ethanol feeding ^1,2^.

Fatty acid (%)	C	CF25	CF57	E	EF25	EF57	Ethanol × Fish Oil
C16:0	20.34 ± 0.8	21.42 ± 0.27	21.05 ± 0.76	16.88 ± 0.48 *	17.05 ± 0.4	15.73 ± 0.5	0.2562
C18:0	20.16 ± 0.82 ^a^	18.72 ± 0.44 ^a,b^	18.33 ± 0.4 ^b^	20.26 ± 0.47	20.5 ± 0.45	21.48 ± 0.91	0.0546
C18:1 (OA, *n*-9)	15.63 ± 1.43 ^a^	11.22 ± 0.49 ^b^	9.15 ± 0.28 ^b^	19.03 ± 0.66 *^,e^	12.25 ± 0.84 ^f^	9.72 ± 0.63 ^g^	0.18
C18:2 (LA, *n*-6)	11.68 ± 0.3 ^a^	11.92 ± 0.34 ^a^	9.25 ± 0.49 ^b^	11.84 ± 0.42 ^e^	9.58 ± 0.3 ^f^	9.29 ± 0.21 ^f^	0.0005
C18:3 (ALA, *n*-3)	0.18 ± 0.01	0.17 ± 0.01	0.18 ± 0.02	0.09 ± 0.03 *	0.19 ± 0.05	0.15 ± 0.05	0.1761
C20:4 (AA, *n*-6)	23.35 ± 1.12 ^a^	15.25 ± 0.37 ^b^	15.9 ± 0.39 ^b^	23.51 ± 0.35 ^e^	14.61 ± 0.57 ^g^	16.78 ± 0.69 ^f^	0.6259
C20:5 (EPA, *n*-3)	0.08 ± 0.02 ^c^	4.07 ± 0.21 ^b^	4.58 ± 0.13 ^a^	0.27 ± 0.29 ^g^	5.24 ± 0.15 ^f^	6.44 ± 0.33 ^e^	0.001
C22:5 (DPA, *n*-3)	0.47 ± 0.06 ^c^	1.81 ± 0.07 ^b^	2.02 ± 0.07 ^a^	0.5 ± 0.07 ^f^	2.9 ± 0.17 ^e^	2.52 ± 0.24 ^e^	0.0002
C22:6 (DHA, *n*-3)	4.8 ± 0.8 ^c^	13.53 ± 0.32 ^b^	17.14 ± 0.35 ^a^	4.4 ± 0.28 ^f^	15.4 ± 0.7 ^e^	15.85 ± 1.01 ^e^	0.0367
SFAs	41.72 ± 0.25 ^a^	40.57 ± 0.48 ^a,b^	39.96 ± 0.69 ^b^	37.51 ± 0.32	38.3 ± 0.55	38.08 ± 1.15	0.1566
MUFAs	16.2 ± 1.51 ^a^	11.52 ± 0.54 ^b^	9.56 ± 0.33 ^b^	19.03 ± 0.66 ^e^	12.25 ± 0.84 ^f^	9.78 ± 0.68 ^g^	0.2783
PUFAs	42.08 ± 1.54 ^b^	47.91 ± 0.31 ^a^	50.48 ± 0.49 ^a^	43.47 ± 0.41 ^g^	49.45 ± 0.69 ^f^	52.13 ± 0.59 ^e^	0.9785
Total *n*-3	5.53 ± 0.85 ^b^	19.59 ± 0.21 ^a^	23.91 ± 0.28 ^a^	5.26 ± 0.52 ^f^	23.73 ± 0.71 ^e^	24.96 ± 1.16 ^e^	0.0054
Total *n*-6	24.86 ± 1.08 ^a^	16.4 ± 0.38 ^b^	17.32 ± 0.41 ^b^	26.37 ± 0.21 ^e^	16.14 ± 0.5 ^g^	17.88 ± 0.73 ^f^	0.3082
*n*-6/*n*-3	4.79 ± 0.48 ^a^	0.84 ± 0.02 ^b^	0.72 ± 0.02 ^b^	5.2 ± 0.45 ^e^	0.68 ± 0.04 ^f^	0.73 ± 0.06 ^f^	0.5105

^1^ Values are expressed as the mean ± SEM. An asterisk (*) shows a significant difference between C and E groups (*p* < 0.05). Means with different superscript letters shows a significant difference (^a,b,c^) among C, CF25, and CF57 groups (*p* < 0.05). Means with different superscript letters shows a significant difference (^e,f,g^) among E, EF25, and EF57 groups (*p* < 0.05). ^2^ Crude lipids of hepatic cell membrane were extracted according to the method of Lee et al. [16]. Details of the fatty-acid methyl ester (FAME) analysis using a capillary gas chromatograph were same as those given in Lee et al. [16]. Details are the same as those described in Table 3. Abbreviations are described in the footnotes to Table 6.

**Table 8:** Correlations of hepatic *n*-3 and *n*-6 fatty acids, the *n*-6/*n*-3 ratio, and histopathology scores with factors of lipid metabolism ^1^.

	Hepatic *n*-3 Fatty Acids		Hepatic *n*-6 Fatty Acids		Hepatic *n*-6/*n*-3 Ratio	
	*r*	*p*	*r*	*p*	*r*	*p*
Steatosis	−0.28714	0.1239	0.37631	0.0404	0.35871	0.0516
Inflammatory cell infiltration	−0.44613	0.00135	0.50184	0.0047	0.50691	0.0043
Adiponectin	0.29971	0.0758	−0.35711	0.0325	−0.34411	0.0399
SIRT1	0.46795	0.004	−0.32857	0.0504	−0.44387	0.0067
MCAD	0.43524	0.008	−0.25395	0.135	−0.32566	0.0526
CPT-1	0.37398	0.0418	−0.1905	0.3133	−0.32736	0.0774

^1^ SIRT1, NAD-dependent deacetylase sirtuin-1; MCAD, medium-chain acyl-coenzyme A dehydrogenase; CPT-1, carnitine palmitoyl transferase I.

## References

[1] Shepherd J. (1994). Violent crime: The role of alcohol and new approaches to the prevention of injury. Alcohol Alcohol..

[2] Anand B.S., Velez M. (2000). Influence of chronic alcohol abuse on hepatitis C virus replication. Dig. Dis..

[3] Umeki S., Shiojiri H., Nozawa Y. (1984). Chronic ethanol administration decreases fatty acyl-CoA desaturase activities in rat liver microsomes. FEBS Lett..

[4] Nanji A.A., Sadrzadeh S.M., Yang E.K., Fogt F., Meydani M., Dannenberg A.J. (1995). Dietary saturated fatty acids: A novel treatment for alcoholic liver disease. Gastroenterology.

[5] Nanji A.A., Griniuviene B., Yacoub L.K., Fogt F., Tahan S.R. (1995). Intercellular adhesion molecule-1 expression in experimental alcoholic liver disease: Relationship to endotoxemia and TNF alpha messenger RNA. Exp. Mol. Pathol..

[6] Nanji A.A., Fogt F., Griniuviene B. (1995). Alterations in glucose transporter proteins in alcoholic liver disease in the rat. Am. J. Pathol..

[7] Chen J.R., Chen Y.L., Peng H.C., Lu Y.A., Chuang H.L., Chang H.Y., Wang H.Y., Su Y.J., Yang S.C. (2016). Fish Oil Reduces Hepatic Injury by Maintaining Normal Intestinal Permeability and Microbiota in Chronic Ethanol-Fed Rats. Gastroenterol. Res. Pract..

[8] Rogers C.Q., Ajmo J.M., You M. (2008). Adiponectin and alcoholic fatty liver disease. IUBMB Life.

[9] You M., Jogasuria A., Taylor C., Wu J. (2015). Sirtuin 1 signaling and alcoholic fatty liver disease. Hepatobiliary Surg. Nutr..

[10] Wada S., Yamazaki T., Kawano Y., Miura S., Ezaki O. (2008). Fish oil fed prior to ethanol administration prevents acute ethanol-induced fatty liver in mice. J. Hepatol..

[11] Lee H.I., Lee M.K. (2015). Coordinated regulation of scopoletin at adipose tissue-liver axis improved alcohol-induced lipid dysmetabolism and inflammation in rats. Toxicol. Lett..

[12] Chiu W.C., Huang Y.L., Chen Y.L., Peng H.C., Liao W.H., Chuang H.L., Chen J.R., Yang S.C. (2015). Synbiotics reduce ethanol-induced hepatic steatosis and inflammation by improving intestinal permeability and microbiota in rats. Food Funct..

[13] Folch J., Lees M., Sloane Stanley G.H. (1957). A simple method for the isolation and purification of total lipides from animal tissues. J. Biol. Chem..

[14] Chen Y.L., Peng H.C., Wang X.D., Yang S.C. (2015). Dietary saturated fatty acids reduce hepatic lipid accumulation but induce fibrotic change in alcohol-fed rats. Hepatobiliary Surg. Nutr..

[15] Zhang H., Li Y., Hu J., Shen W.J., Singh M., Hou X., Bittner A., Bittner S., Cortez Y., Tabassum J. (2015). Effect of Creosote Bush-Derived NDGA on Expression of Genes Involved in Lipid Metabolism in Liver of High-Fructose Fed Rats: Relevance to NDGA Amelioration of Hypertriglyceridemia and Hepatic Steatosis. PLoS ONE.

[16] Lee H.C., Liang A., Lin Y.H., Guo Y.R., Huang S.Y. (2017). Low dietary n-6/n-3 polyunsaturated fatty acid ratio prevents induced oral carcinoma in a hamster pouch model. Prostaglandins Leukot. Essent. Fatty Acids.

[17] Mezey E. (1991). Interaction between alcohol and nutrition in the pathogenesis of alcoholic liver disease. Semin. Liver Dis..

[18] Israel Y., Britton R.S., Orrego H. (1982). Liver cell enlargement induced by chronic alcohol consumption: Studies on its causes and consequences. Clin. Biochem..

[19] Li Q., Zhong W., Qiu Y., Kang X., Sun X., Tan X., Zhao Y., Sun X., Jia W., Zhou Z. (2013). Preservation of hepatocyte nuclear factor-4alpha contributes to the beneficial effect of dietary medium chain triglyceride on alcohol-induced hepatic lipid dyshomeostasis in rats. Alcohol. Clin. Exp. Res..

[20] Gao B., Bataller R. (2011). Alcoholic liver disease: Pathogenesis and new therapeutic targets. Gastroenterology.

[21] Yuan F., Wang H., Tian Y., Li Q., He L., Li N., Liu Z. (2016). Fish oil alleviated high-fat diet-induced non-alcoholic fatty liver disease via regulating hepatic lipids metabolism and metaflammation: A transcriptomic study. Lipids Health Dis..

[22] Zelber-Sagi S., Salomone F., Mlynarsky L. (2017). The Mediterranean dietary pattern as the diet of choice for non-alcoholic fatty liver disease: Evidence and plausible mechanisms. Liver Int..

[23] Ajmo J.M., Liang X., Rogers C.Q., Pennock B., You M. (2008). Resveratrol alleviates alcoholic fatty liver in mice. Am. J. Physiol. Gastrointest. Liver Physiol..

[24] Jiang Z., Zhou J., Zhou D., Zhu Z., Sun L., Nanji A.A. (2015). The adiponectin-SIRT1-AMPK pathway in alcoholic fatty liver disease in the rat. Alcohol. Clin. Exp. Res..

[25] You M., Liang X., Ajmo J.M., Ness G.C. (2008). Involvement of mammalian sirtuin 1 in the action of ethanol in the liver. Am. J. Physiol. Gastrointest. Liver Physiol..

[26] Qin Y., Zhou Y., Chen S.H., Zhao X.L., Ran L., Zeng X.L., Wu Y., Chen J.L., Kang C., Shu F.R. (2015). Fish Oil Supplements Lower Serum Lipids and Glucose in Correlation with a Reduction in Plasma Fibroblast Growth Factor 21 and Prostaglandin E2 in Nonalcoholic Fatty Liver Disease Associated with Hyperlipidemia: A Randomized Clinical Trial. PLoS ONE.

[27] Hosoyamada Y., Yamada M. (2017). Effects of Dietary Fish Oil and Apple Polyphenol on the Concentration Serum Lipids and Excretion of Fecal Bile Acids in Rats. J. Nutr. Sci. Vitaminol. (Tokyo).

[28] Xu A., Wang Y., Keshaw H., Xu L.Y., Lam K.S., Cooper G.J. (2003). The fat-derived hormone adiponectin alleviates alcoholic and nonalcoholic fatty liver diseases in mice. J. Clin. Investig..

[29] Shen Z., Liang X., Rogers C.Q., Rideout D., You M. (2010). Involvement of adiponectin-SIRT1-AMPK signaling in the protective action of rosiglitazone against alcoholic fatty liver in mice. Am. J. Physiol. Gastrointest. Liver Physiol..

[30] Nakatani T., Kim H.J., Kaburagi Y., Yasuda K., Ezaki O. (2003). A low fish oil inhibits SREBP-1 proteolytic cascade, while a high-fish-oil feeding decreases SREBP-1 mRNA in mice liver: Relationship to anti-obesity. J. Lipid Res..

[31] Atek-Mebarki F., Hichami A., Abdoul-Azize S., Bitam A., Koceir E.A., Khan N.A. (2015). Eicosapentaenoic acid modulates fatty acid metabolism and inflammation in Psammomys obesus. Biochimie.

[32] Wood C.B., Habib N.A., Thompson A., Bradpiece H., Smadja C., Hershman M., Barker W., Apostolov K. (1985). Increase of oleic acid in erythrocytes associated with malignancies. Br. Med. J. (Clin. Res. Ed.).

[33] Pala V., Krogh V., Muti P., Chajes V., Riboli E., Micheli A., Saadatian M., Sieri S., Berrino F. (2001). Erythrocyte membrane fatty acids and subsequent breast cancer: A prospective Italian study. J. Natl. Cancer Inst..

[34] Kennedy A., Martinez K., Chuang C.C., LaPoint K., McIntosh M. (2009). Saturated fatty acid-mediated inflammation and insulin resistance in adipose tissue: Mechanisms of action and implications. J. Nutr..

[35] Yamada K., Mizukoshi E., Sunagozaka H., Arai K., Yamashita T., Takeshita Y., Misu H., Takamura T., Kitamura S., Zen Y. (2015). Characteristics of hepatic fatty acid compositions in patients with nonalcoholic steatohepatitis. Liver Int..

[36] Visscher C., Middendorf L., Gunther R., Engels A., Leibfacher C., Mohle H., Dungelhoef K., Weier S., Haider W., Radko D. (2017). Fat content, fatty acid pattern and iron content in livers of turkeys with hepatic lipidosis. Lipids Health Dis..

